# An efficient semi-supervised community detection framework in social networks

**DOI:** 10.1371/journal.pone.0178046

**Published:** 2017-05-23

**Authors:** Zhen Li, Yong Gong, Zhisong Pan, Guyu Hu

**Affiliations:** College of Command Information Systems, PLA University of Science & Technology, Nanjing, Jiangsu, China; Universitat Rovira i Virgili, SPAIN

## Abstract

Community detection is an important tasks across a number of research fields including social science, biology, and physics. In the real world, topology information alone is often inadequate to accurately find out community structure due to its sparsity and noise. The potential useful prior information such as pairwise constraints which contain must-link and cannot-link constraints can be obtained from domain knowledge in many applications. Thus, combining network topology with prior information to improve the community detection accuracy is promising. Previous methods mainly utilize the must-link constraints while cannot make full use of cannot-link constraints. In this paper, we propose a semi-supervised community detection framework which can effectively incorporate two types of pairwise constraints into the detection process. Particularly, must-link and cannot-link constraints are represented as positive and negative links, and we encode them by adding different graph regularization terms to penalize closeness of the nodes. Experiments on multiple real-world datasets show that the proposed framework significantly improves the accuracy of community detection.

## Introduction

In many different disciplines, relations exist in the form of networks, such as social networks [1–3] and biological networks [4]. These networks usually exhibit a strong community structure which are clusters whose nodes are more tightly connected with each other than with nodes outside the cluster [5]. Community detection is critical for better understanding the networks especially huge networks, which enables us to observe and analyze the networks from community level. It also promotes other related social computing tasks such as recommendation systems [6]. For example, in terms of a community of users with similar interests, the operator can recommend relevant goods to a user according to the consumptions of other users in the same community. Community detection provides complementary basis for recommendation system which is based on the users’ past preferences, helps operators to provide better service. Thus, community detection has attracted great attention.

During the past years, a large number of community detection algorithms have been proposed [1–3,5–7], most of them are based on the topology information [1,5–7]. However, topology information alone is inadequate to accurately derive community structure for two reasons. Firstly, due to the complexity of network structure, such as overlapping communities or hierarchical structures, many traditional methods will degrade when community structure is not clear [8,9]. Secondly, it is hard to accurately detect the community due to the sparsity and noises of the topology information [10–12]. Works in [8, 9] show that there is a threshold on the difference between intra and inter community edge number, below which communities are impossible for any algorithm to detect, which is named as community detectability. Many community detection algorithms, such as spectral methods, succeed when the network is sufficiently dense [9], and they fail significantly when the number of external edges between communities increases [8]. In many real scenarios, some related prior information can be gained in advance. The prior information is often in the form of pairwise constraints which contains must-link and cannot-link constraints [10]:

- *must-link constraints*: the two nodes should belong to the same community.
- *cannot-link constraints*: the two nodes should belong to different communities.

Several methods designed for unsigned networks have been proposed to consider the prior information [10–17], which are named as semi-supervised community detection. It has been proved that the accuracy and robustness will be significantly improved with the prior information, especially on most real-world complicated and noisy networks. Thus, it is promising to incorporate the prior information to guide the community detection process.

However, most of the above methods are developed to make full use of must-link constraints while little works concentrate on utilizing the cannot-link constraints. Must-link and cannot-links constraints are usually transferred to the connect edges and disconnect edges in the network. Cannot-link nodes that modeled to be not connected with each other can also be in the same community, while they should be in different communities. In this paper, we represent the two types of prior information as positive and negative links. In many real-world systems, the relations between users are various which include not only positive relations like friendships, trusts and likes, but also negative relations like antagonisms, distrusts and dislike. In the social network analysis field, positive and negative links are used to represent the above two types of relations respectively [18], the corresponding network is usually called signed network [18, 19]. Here we transfer the must-link and cannot-link constraints into positive and negative links respectively to make full use of them. Due to the intrinsic property of the pairwise constraints, the communities in the network with both positive and negative links are the groups that (1) within which the negative links are sparse and (2) between which the positive links are also sparse [18]. Then nodes restricted with must-link constraints are assigned to the same community while with cannot-link constraints are assigned to different communities.

In this paper, we propose an efficient semi-supervised community detection framework based on Nonnegative Matrix Factorization (NMF), which combines the prior information with the topology to extract high-quality communities. The main contributions in this paper are summarized as follow:

1. We transfer must-link and cannot-link constraints into positive and negative links respectively, and build a signed network. Then we develop a method to map the signed network from high-dimensional space to low-dimensional space based on the strategies of mining communities in signed networks. Particularly, two types of prior information are separately utilized by introducing different graph regularizations to the NMF objective function, then both the types of prior information can be fully utilized.
2. The semi-supervised framework we develop is flexible, in which the two graph regularizations can be introduced to other basic community detection algorithms such as spectral clustering algorithm to guide the detection process with prior information.
3. Experiments are carried out on seven real-world datasets, and the results demonstrate the effectiveness of our method.

The rest of this paper is organized below: In Related work section, we review the related work briefly. In the following section, we introduce the basic problem definition and the related notions, then we describe our method in details. Experimental results on real-world data are presented in Experiments section. Finally, we make some conclusions.

## Related work

Many semi-supervised community detection methods for unsigned networks have been proposed in the past few years [10–17]. Wang et al. [10] proposed a matrix factorization based approach for semi-supervised clustering, and extended it to co-cluster the multiple type data points with different types of constraints simultaneously. Allahverdyan et al. [11] studied the problem of semi-supervised graph clustering by integrating known cluster assignments for a fraction of nodes. However, the prior information considered in the two papers was the labels of some nodes rather than pairwise constraints. In fact, the latter type of prior information is more realistic in real world because it is easier to assess similarity of two objects rather than to label them individually [12]. And it is more difficult to process pairwise constraints because only whether a pair of nodes belong to the same community is known rather than the labels. Steeg et al. [12] examined the impact of pairwise constraints on the clustering accuracy from the perspective of statistical mechanics. However, only the network composed of two equal-sized communities was studied, while in real world most networks are composed of several communities of different sizes. Eaton et al. [13] utilized a spin-glass model from statistical physics, which was a generalization of modularity Q function, to combine prior knowledge in forms of individual labels and pairwise constrains by penalizing for community structures that violate the guidance. However, the performance of the method deteriorates sometimes when there are many cannot-link priors. Ma et al. [14] encoded the must-link and cannot-link constraints into the adjacency matrix and factorized it to get the indicator matrix. In the paper, the prior information was processed together with and in the same way as the original network topology, however, it contains more definite information and deserves separate efforts. Zhang et al. [15] encoded the prior information by directly modified the adjacency matrix in which must-link and cannot-link constraints were modeled as connect and disconnect edges, and added a logical inference step to better utilize the two types of prior information. In this paper the prior information was used by transferring and modifying the adjacency matrix directly. However, the cannot-link constraints were not fully utilized because disconnect edges have no definite indications. Yang et al. [16] firstly presented a unified interpretation to a group of existing community detection methods, then proposed a unified semi-supervised framework to integrate network topology with prior information based on this interpretation. They encoded must-link constraints by adding a graph regularization term to penalize the latent space dissimilarity of these nodes. However, cannot-link constraints were not considered in this paper. Wang et al. [17] proposed a semi-supervised framework which constructed a matrix from the positive and negative labels to fully utilize the prior information. The authors also investigated the effect of the priors on the community detection. But the algorithm is complex and has a poor performance in large size networks.

The authors in paper [15] have evaluated which type of pairwise prior is more useful, and find that constraints of must-link contribute more than that of cannot-link. Though cannot-link priors are less useful, in some cases only this type of prior information is available, and we should make efforts to fully utilize them. Though many semi-supervised community detection methods are developed, most of them do not effectively use the cannot-link constraints to guide the detecting process. How to make better use of the prior knowledge especially cannot-link prior is still a question. In the existing papers, the must-link constraints and cannot-link constraints are mostly modeled as connected edges and disconnect edges between nodes respectively, that is there is an edge between must-link nodes and no edge between cannot-link nodes. Then the must-link edge are dealt with separately while cannot-link constraints are not utilized because disconnect edges do not definitely indicate the membership of the nodes. However, actually cannot-link constraints strongly indicates that the nodes belong to different communities. In this paper we model the cannot-link constraints as negative links and construct a signed network, then we propose a semi-supervised community detection framework based on the rule that between communities negative links should be dense while positive links should be sparse, the framework can incorporate cannot-link constraints more effectively. In this paper, we develop the framework based on NMF. NMF [20] is a popular matrix factorization method where all the elements related are restricted to be nonnegative, it performs well in social network analysis field [21–23]. In this paper, we propose a semi-supervised community detection algorithm in which two graph regularization terms are introduced to penalize the membership of the nodes to fully utilize the pairwise constraints. The framework maps the original network to a new lower-dimensional space, where the original underlying structure information is more explicit and clear.

## Semi-supervised community detection framework

### Notations

The notations used in this paper are firstly introduced, which are shown in Table 1. In terms of a signed network, *n* and *k* are used to denote the number of nodes and the number of communities respectively. *A* ∈ *R*^*n*×*n*^ is the adjacency matrix of the original network where *A*_*ij*_ = 1 and *A*_*ij*_ = 0 denote a link and no link between node *i* and node *j* respectively. The matrix *A*^*p*^ ∈ *R*^*n*×*n*^ represents a positive network where $A_{ij}^{p}=1$ denotes a positive link between node *i* and node *j* and $A_{ij}^{p}=0$ otherwise. Similarly, *A*^*n*^ ∈ *R*^*n*×*n*^ is used to represent a negative network where $A_{ij}^{n}=1$ denotes a negative link and $A_{ij}^{n}=0$ otherwise. In this paper, we transfer the must-link and cannot-link constraints into positive and negative links respectively, then the corresponding adjacency matrices *A*^*p*^ and *A*^*n*^ can be gained. The matrix *H* ∈ *R*^*n*×*k*^ is the clustering indicator, where *H*_*ij*_ denotes the strength of node *i* belonging to *j*-th cluster. Normally, it is thought that node *i* belongs to the *m*-th community if *H*_*im*_ is the max value of *H*(*i*,:).

**Table 1:** Notations definitions.

**Notations**	**Explanations**	**Dimension**
*n*	number of nodes	-
*k*	number of communities	-
*A*	the adjacency matrix of the original network	*n*×*n*
*A^p^*	the adjacency matrix of a positive network	*n*×*n*
*A^n^*	the adjacency matrix of a negative network	*n*×*n*
*H*	clustering indicator matrix	*n*×*k*
*L*	Laplacian matrix	*n*×*n*

### Problem statement

Given a network and prior information, we represent the must-link and cannot-link constraints as positive and negative links respectively. Then we can build the positive and negative networks, whose nodes are of the same set as the original network and link are gained from the must-link and cannot-link constraints. The links of the original unsigned network are processed as positive links. With the notations given above, the semi-supervised community detection problem in this paper is formally defined as follows:

#### Problem 1

*Given a network G = (V*, *E) with adjacency matrix A*, *a positive network with adjacency matrix Ap and a negative network with adjacency matrix An*, *find k communities* {*C*_*1*_,*…C*_*k*_} *with maximal positive links within communities and maximal negative links across communities, i.e., $\max\sum_{i,j}A_{ij}\delta (c_{i},c_{j})$, $\max\sum_{i,j}A_{ij}^{p}\delta (c_{i},c_{j})$ and $\max\sum_{i,j}A_{ij}^{n}(1-\delta (c_{i},c_{j}))$, where δ*(*c*_*i*_,*c*_*j*_) *is the Kronecker delta function which is 1 if node i and node j belong to the same community*, *0 otherwise*.

In terms of this problem, we attempt to develop a reasonable matrix factorization method to approximate the adjacency matrix, which maps the network into a lower-dimensional space to make the indicated structure information explicit. Besides, we introduce two graph regularization terms to push the nodes with must-link constraints into the same communities while those with cannot-link constraints into different communities.

### Proposed method

In this subsection, the proposed framework based on NMF with different types of regularizations is presented.

In paper [22], the 2-factor NMF *A* ≈ *WH*^*T*^ has been adopted to find communities where *H* is the community indicator which denotes the likelihood that node *i* belongs to the *j*-th community. NMF has been proved useful to capture node similarity and mutual influences between links. In terms of undirected networks, the adjacency matrices are symmetric and then the symmetric NMF is developed, i.e., *A* ≈ *HH*^*T*^ [21]. In this paper, we focus on undirected networks and use *H* to decide the nodes’ membership. From the viewpoint of clustering, we can regard the factorization process as projecting the *n* dimension feature in the adjacency matrix into a *k* dimension latent space. The objective function of NMF is as follows:

$$\underset{H\in R^{n\times k}}{\min}{\left\|A-HH^{T}\right\|}_{F}^{2}\;s.t.H_{ij}\geq 0,\forall i,j$$ (1)

where *A* is the adjacency matrix of the original network, ‖⋅‖_*F*_ denotes the matrix Frobenius norm which is defined as ${\left\|A\right\|}_{F}=\sqrt{\sum_{i=1}^{m}\sum_{j=1}^{n}|A_{ij}{|}^{2}}$.

In order to keep negative links between communities to enforce the nodes with cannot-link constraints to be in different communities, we introduce a graph regularization to minimize the number of negative links inside each community relative to its size. In particular, we define a indicator matrix to describe the nodes’ membership, which is composed of *k* indicator vectors, i.e. *H* = [*h*_1_,…*h*_*k*_]. The regularization is defined as follows:

$$\min\sum_{c=1}^{k}\frac{h_{c}^{T}A^{n}h_{c}}{h_{c}^{T}h_{c}}$$ (2)

where *A*^*n*^ is the adjacency matrix of a negative network, *π*_*c*_ denotes the *c*-th community, and *h*_*c*_ is an *n*-dimensional vector which denotes whether a node belongs to *c*-th community. If node *i* belongs to *π*_*c*_, the value of *h*_*c*_(*i*) is 1, otherwise, *h*_*c*_(*i*) = 0. Note that we study the non-overlapping cases in this paper, i.e., *h*_*c*_s are subject to that each node must be a member of one and only one community. The regularization enforces the nodes connected with negative links to be distributed into different communities, then the goal of keeping negative links between communities dense can be achieved. Because the optimization problem (2) is an NP-hard problem, in this paper, we solve this problem by relaxing *h*_*c*_ to be continuous. Then the indicator vector *h*_*c*_ has the following form:

$$h_{c}(i)=\left\{ \begin{array}{l} \sqrt{1/|\pi_{c}|},node\;i\in \pi_{c}\\ \;0,\;otherwise \end{array}\right.$$ (3)

with |*π*_*c*_|>0,*c* = 1,…*k*. We observe that $tr(H^{T}A^{n}H)=\sum_{c=1}^{k}\frac{h_{c}^{T}A^{n}h_{c}}{h_{c}^{T}h_{c}}$. Then the solution to the optimization problem (2) can be obtained by addressing the following optimization problem:

$$\underset{H\in R^{n\times k}}{\min}tr(H^{T}A^{n}H)\;s.t.H_{ij}\geq 0,\forall i,j$$ (4)

Similarly, we introduce a graph regularization to minimize the number of positive links between communities relative to its size [6]. The regularization is defined as follows:

$$\min\sum_{c=1}^{k}\frac{h_{c}^{T}(D-A^{p})h_{c}}{h_{c}^{T}h_{c}}=\min\sum_{c=1}^{k}\frac{h_{c}^{T}Lh_{c}}{h_{c}^{T}h_{c}}$$ (5)

where *D* is the diagonal matrix whose entries are row summation of *A*^*p*^, *L = D-A*^*p*^ is the Laplacian matrix of *A*^*p*^. Follow the method of constructing the negative links regularization, we obtain the function as follows:

$$\underset{H\in R^{n\times k}}{\min}tr(H^{T}LH)\;s.t.H_{ij}\geq 0,\forall i,j$$ (6)

Combining the two types of graph regularizations, the final optimization objective we develop is defined as Formula (7):

$$\underset{H\in R^{n\times k}}{\min}{\left\|A-HH^{T}\right\|}_{F}^{2}+\gamma_{1}tr(H^{T}A^{n}H)+\gamma_{2}tr(H^{T}LH)\;s.t.H_{ij}\geq 0,\forall i,j$$ (7)

where *γ*_1_,*γ*_2_ are parameters to control the weights of the graph regularizations which act as penalizations to push the constrained nodes into correct communities.

### The updating rules

In this section, we discuss how to solve the optimization problem (7). The optimal solution can be achieved using the iterative update algorithm we develop. Let *α* be the Lagrange matrix for constraint *H* ≥ 0, then the Lagrangian function can be written as:

$$\begin{array}{l} l={\left\|A-HH^{T}\right\|}_{F}^{2}+\gamma_{1}tr(H^{T}A^{n}H)+\gamma_{2}tr(H^{T}LH)-\alpha H\\ \;=tr((A-HH^{T}){\left(A-HH^{T}\right)}^{T})+\gamma_{1}tr(H^{T}A^{n}H)+\gamma_{2}tr(H^{T}LH)-\alpha H\\ \;=tr(AA^{T})-2tr(AHH^{T})+tr(HH^{T}HH^{T})+\gamma_{1}tr(H^{T}A^{n}H)+\gamma_{2}tr(H^{T}LH)-\alpha H \end{array}$$ (8)

Then the derivative of *l* with respect to *H* is:

$$\frac{\partial l}{\partial H}=-4AH+4HH^{T}H+2\gamma_{1}A^{n}H+2\gamma_{2}LH-\alpha$$ (9)

According to [22], using the KKT condition (−4*AH* + 4*HH*^*T*^ *H* + 2*γ*_1_*A*^*n*^*H* + 2*γ*_2_*LH*)_*ij*_*H*_*ij*_ = 0, which is the fixed point relation that *H* must hold, and because *H* ≥ 0, we have that ${\left(-4AH+4HH^{T}H+2\gamma_{1}A^{n}H+2\gamma_{2}LH\right)}_{ij}H_{ij}^{2}=0$. Then given an initial guess of *H*, the updating rule is shown as follows:

$$H_{ij}\leftarrow H_{ij}\sqrt{\frac{{\left(2AH+\gamma_{2}A^{p}H\right)}_{ij}}{{\left(2HH^{T}H+\gamma_{1}A^{n}H+\gamma_{2}DH\right)}_{ij}}}$$ (10)

With the updating rule, the optimization algorithm is presented in Algorithm 1. Note that we update one matrix while fixing the other matrices in each step and the iterative process is stopped if these cluster matrix converges or the number of iterative reaches a given threshold. As the cost of each term in Formula (10) is *O*(*n*^2^*k*), then the time complexity of updating rule in each iteration is *O*(*n*^2^*k*), which is the same as that of basic symmetric NMF.

**Algorithm 1.**Semi-supervised Community Detection Framework

**Input:** number of communities *k*, adjacency matrix *A*, *A*^*p*^ and *A*^*n*^, parameters: *γ*_1_ and *γ*_2_

**Output:** communities *C*_1_,*C*_2_……*C*_*k*_

1: Initialize *H* ≥ 0

2: **while** not convergent **do**

3: update *H* according to Formula (10)

4: **end while**

5: obtaining *C*_*j*_ from *H* by finding the max value and its index in each row of *H*.

## Experiments

To test the performance of the proposed semi-supervised community detection framework, we verify the performance improvement on some widely used real-world networks, and compare it with three existing semi-supervised community detection methods.

### Datasets

The basic information of the datasets used in our experiments is described as follows. Table 2 summarizes the basic information of those datasets.

- **Karate** (https://doi.org/10.6084/m9.figshare.4993895.v1). This is the well-known Zachary karate club network. The data was collected from the members of a university karate club by Wayne Zachary in 1977. Each node represents a member of the club, and each edge represents a tie between two members of the club. The members are split into two groups after an argument between two teachers.
- **Dolphins** (https://doi.org/10.6084/m9.figshare.4993895.v1). This is a social network of bottlenose dolphins. The nodes are the bottlenose dolphins living off Doubtful Sound. An edge indicates a frequent association. It contains 62 dolphins which are classified into 2 communities.
- **Football** (https://doi.org/10.6084/m9.figshare.4993895.v1). Network of American football games between Division IA colleges during regular season Fall 2000. Nodes in the network represent teams and links represent regular-season games between the two teams they connect. The teams are divided into conferences containing around 8–12 teams each.
- **Polbooks** (https://doi.org/10.6084/m9.figshare.4993895.v1). A network of books about US politics published around the time of the 2004 presidential election and sold by the online bookseller Amazon.com. Edges between books represent frequent co-purchasing of books by the same buyers. According to the political viewpoint, the books are clustered into three categories: ‘liberal’, ‘neutral’, or ‘conservative’.
- **Polblogs** (https://doi.org/10.6084/m9.figshare.4993895.v1). A network of hyperlinks between weblogs on US Presidential Election of 2004, recorded in 2005 by Adamic and Glance. The weblogs are clustered to two communities, i.e., liberal community and conservative community.
- **Politics-uk** (https://doi.org/10.6084/m9.figshare.4993895.v1). This dataset is a collection of 419 Members of Parliament in the UK, and it consists of 5 communities, corresponding to the political parties. There are several networks describing different relations between the same set of users in the dataset, here we choose to use *follows* describing the follow relationship.
- **Olympics** (https://doi.org/10.6084/m9.figshare.4993895.v1). This dataset contains a collection of 464 users which consists of the athletes and organizations involved in the London 2012 Summer Olympics, they are assigned to 28 communities according to different sports. There are several networks describing different relations between the same set of users in the dataset, here we choose to use *follows* describing the follow relationship.

**Table 2:** Details of the networks.

Datasets	Nodes	Links	Communities
**Karate**	34	78	2
**Dolphins**	62	159	2
**Football**	115	613	12
**Polbooks**	105	441	3
**Polblogs**	1490	16718	2
**Politics-uk**	419	19950	5
**Olympics**	464	7787	28

### Evaluation measures and baseline methods

In this paper, we apply Normalized Mutual Information (NMI) [24] and Purity [25] to evaluate the performances of different algorithms. Both NMI and Purity adopt the ground truth as a baseline, the values of the two measures range from 0 to 1 and the higher value means better performance.

NMI estimates the similarity between the ground truth communities and the detected. Let *C* and *G* denote our partition the ground truth respectively, *F* be the confusion matrix whose element *F*_*ij*_ is the number of nodes in community *i* of the partition *C* that are also in the community *j* of the partition *G*, then NMI is defined as follows:

$$NMI(C,G)=\frac{-2\sum_{i=1}^{f_{C}}\sum_{j=1}^{f_{G}}F_{ij}\log(F_{ij}n/F_{i\cdot }F_{\cdot j})}{\sum_{i=1}^{f_{C}}F_{i\cdot }\log(F_{i\cdot }/n)+\sum_{j=1}^{f_{G}}F_{\cdot j}\log(F_{\cdot j}/n)}$$ (11)

where *f*_*C*_ (*f*_*G*_) is the number of communities in the partition *C* (*G*), *F*_*i*⋅_ (*F*_⋅*j*_) is the sum of the elements of *F* in row *i* (column *j*), and *n* is the number of nodes. If *C* = *G*, *NMI(C*, *G)* = 1. If *C* and *G* are completely different, *NMI(C*, *G)* = 0.

Purity is measured by computing the number of nodes assigned with the same labels in all communities. Let *G* = {*G*_1_,…*G*_*k*_} be the set of communities in the ground truth and *C* = {*C*_1_,…*C*_*t*_} be the *t* communities extracted by different approaches. Purity is defined as:

$$Purity=\frac{1}{n}\sum_{i=1}^{t}\underset{j}{\max}|C_{i}\cap G_{j}|$$ (12)

where *n* is the number of nodes.

In order to measure the effectiveness of the semi-supervised framework, three existing methods, i.e., the NMF_LSE (Loss of Square or Euclidean function based NMF) based algorithm [16] and PMF (Penalized Matrix Factorization) based algorithm [17], and SNMF_SS (Symmetric NMF based Semi-Supervised clustering) based algorithm [14] are compared in our experiments.

- NMF_LSE-based algorithm. In paper [16], Yang et al. propose a unified semi-supervised framework to integrate network topology with must-link prior information for community detection, which encodes the prior information by adding a graph regularization term *tr*(*H*^*T*^ *LH*) to penalize the latent space dissimilarity of these nodes. This framework can be applied to many widely-used matrix-based community detection methods satisfying the interpretation, such as NMF, spectral clustering. In this paper, NMF_LSE based framework is chosen as the baseline method, because the main difference between it and our method is how to utilize the prior information. In the experiment, the parameter is set to 1 as suggested by the authors.
- PMF-based algorithm. In paper [17], a PMF-based framework to cluster the multiple type data points simultaneously is proposed. The must-link and cannot-link prior information is dealt with simultaneously by the introducing a graph regularization term *tr*(*H*^*T*^ Θ*H*) where Θ = *δ*, Θ = −*θ*, and Θ = 0 if there is cannot-link constraint, must-link constraint, and no constraint between node *i* and *j*, and *δ*, *θ* are the penalties for violating the cannot-link constraint and must-link constraint respectively. Following the suggestions of the authors, the values of *δ* and *θ* are set to 2 and 1 respectively.
- SNMF_SS-based algorithm. In paper [14], semi-supervised framework based on SNMF which modifies the adjacency matrix with the prior information is proposed. Particularly, adjacency matrix with must-link and cannot-link constraints is developed as: $\tilde{A}=A-\alpha M+\beta C$ where *M* and *C* are the constraint matrices. In the experiment, the parameters *α* and *β* are set to 0.001 and 0.1 as suggested by the authors.

### Experimental results on real-world datasets

There are totally *N*(*N-1*)*/2* pairs of membership in a undirected network with *N* nodes, while the max number of must-link pairs is

$$M_{pairs}=\sum_{k=1}^{K}N_{k}(N_{k}-1)/2$$ (13)

where *K* is the number of the communities and *N*_*k*_ is the number of the nodes in the *k*-th ground-truth community. The percentage of must-link constraints we used in this paper is based on the *M*_*pairs*_. Similarly, the max number of cannot-link pairs is

$$C_{pairs}=\sum_{i=1,i<j}^{K}\sum_{j=2}^{K}N_{i}N_{j}$$ (14)

The percentage of cannot-link constraints in this paper is based on the *C*_*pairs*_.

**Fig 1:**
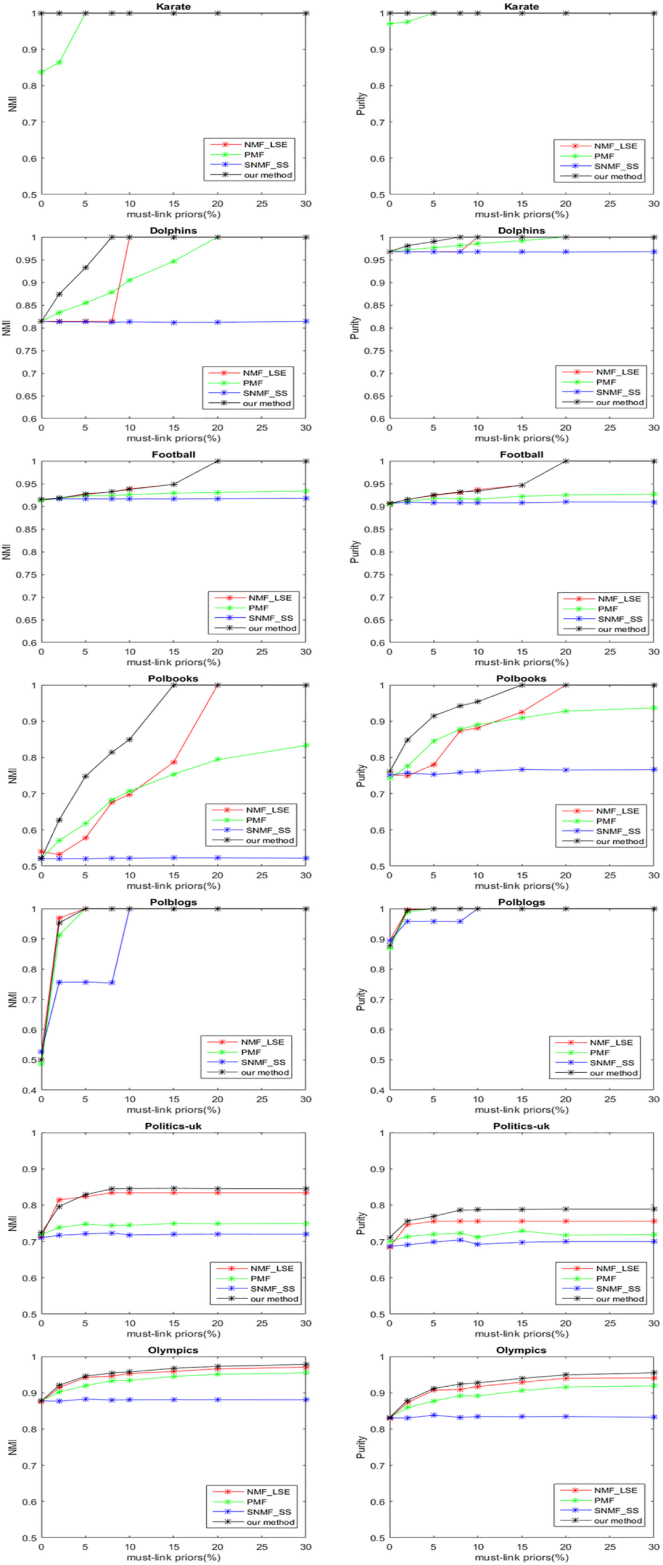
Experimental results of different algorithms with different percentages of must-link priors on seven real-world datasets.

In the experiments, all the results are averaged over 50 different runs and the variance are not larger than 0.01. The regularization parameters of our method are set to 1 for simplicity. And the parameters of baseline methods are set as authors suggested to achieve the best performance. We first conduct the experiments with varying percentages of must-link priors and no cannot-link priors. The performances of all the semi-supervised algorithms on different datasets are shown in Fig 1. Note that in some places the algorithms have the same performance and the lines are overlapping. The NMI and Purity of most methods increase consistently as the used priors increase. Some of them reach 1 when prior information is adequate. It can be observed that various methods have different growth trends, and our method outperforms the others in most cases. Take Polbooks dataset for example, with the same percentage (<15%) of must-link priors, our method performs best among all the algorithms. NMI and Purity of our method reach 1 when the prior percentage is 15%, while NMF_LSE needs 20% priors to have the same performance, PMF and SNMF_SS cannot detect completely correct communities even with 30% priors. The experimental results indicate that our method efficiently utilize must-link priors, the reasons may be as follows: Firstly, we introduce a regularization term to separately deal with must-link priors and the term helps to distribute the constrained nodes into correct communities. Secondly, we adopt the symmetric NMF in the model which can derive more precise information from undirected networks.

**Fig 2:**
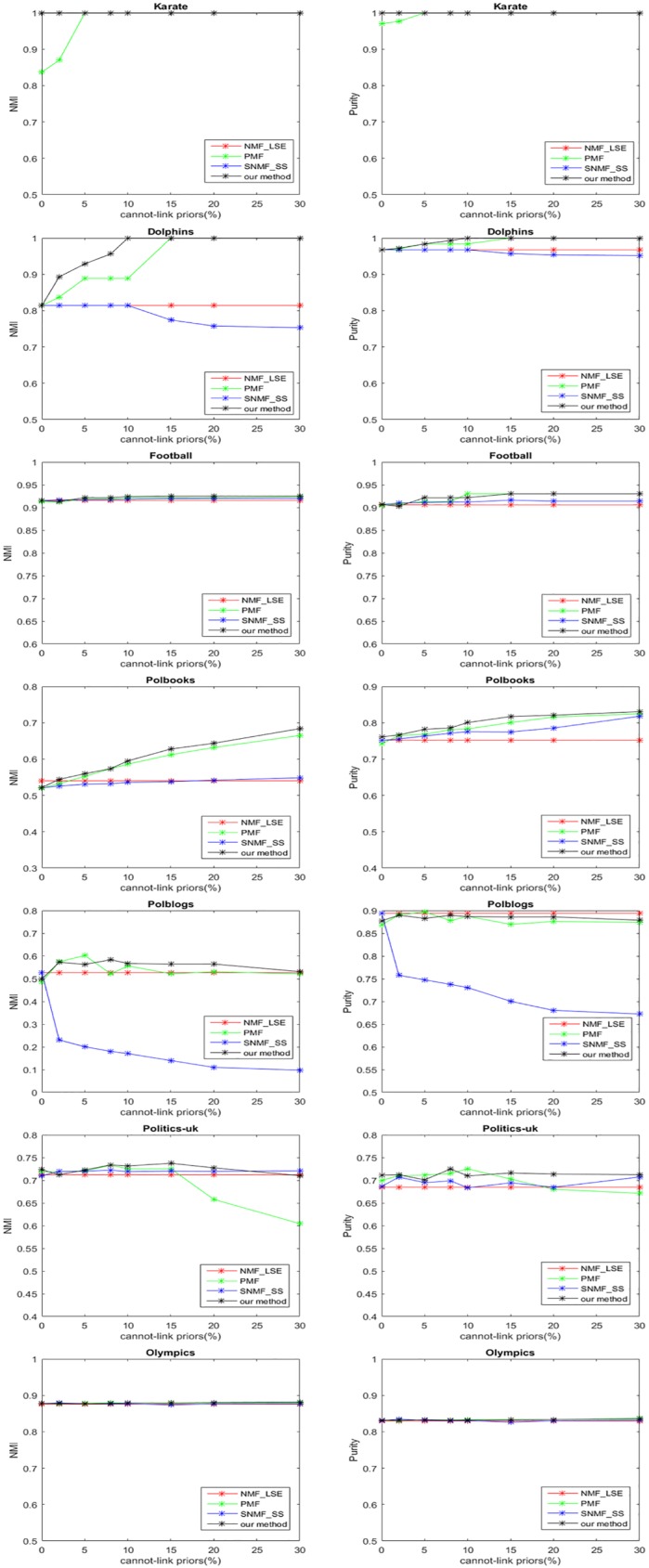
Experimental results of different algorithms with different percentages of cannot-link priors on seven real-world datasets.

Then we evaluate our method on the datasets with varying percentages of cannot-link priors and no must-link priors. The performances of different methods are shown in Fig 2. Note that in some places the algorithms have the same performance and the lines are overlapping. We can see that on Dolphins, Football and Polbooks datasets, NMI and Purity of different method continue to increase as the percentage of prior increases, and our method performances best. The cannot-link priors have no effect on the results of NMF_LSE method because the priors are modeled as disconnect edges in the network. With regard to the other two methods, they deal with cannot-link priors in the same way as must-link priors. However, our method introduce a different regularization term to restrict the nodes to be in different communities so that make better use of cannot-link priors. We can also observe that on Polblogs, Politics-uk and Olympic datasets, the performances do not have significant increase and some even degrade with increasing cannot-link priors. This may be because the number of cannot-link priors is huge in these datasets, and even larger than the number of the links in the original networks. For example, there are 27742 negative links in Polblogs if we add 5% cannot-link priors. Then the detecting process may rely more on the added negative links than the original positive links, and may lose the balance of maximizing positive links within communities and maximizing negative links across communities, furthermore, the forming of communities may fail due to massive negative links and inadequate positive links. So we suggest that cannot-link priors should not be too many, less than original links is better.

**Fig 3:**
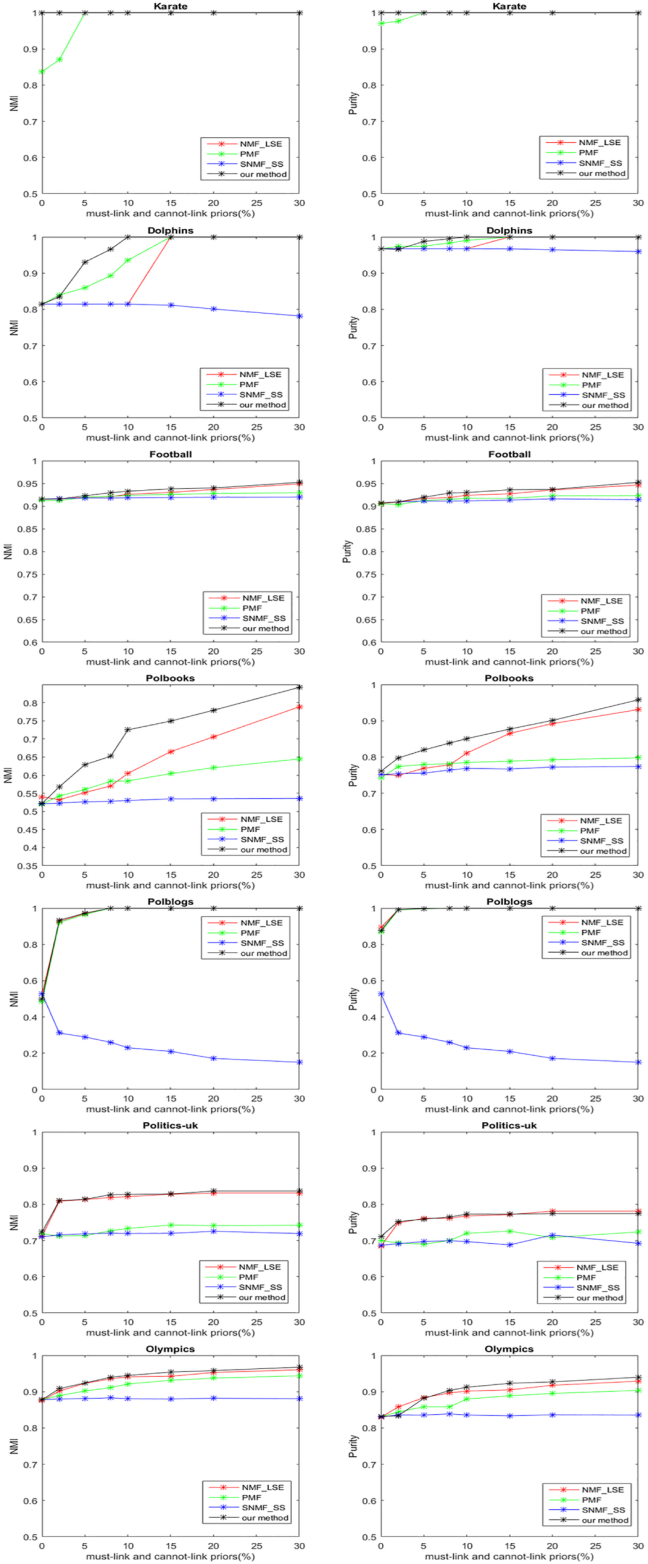
Experimental results of different algorithms with different percentages of priors. Half of the priors are must-link priors and the other half are cannot-link priors.

The experiments with both must-link priors and cannot-link priors are also conducted. The performances are shown in Fig 3. Note that in some places the algorithms have the same performance and the lines are overlapping. In each experiment, half the priors are must-link and the other half are cannot-link priors. We can observe that NMI and Purity of our method increase significantly with the increase of used priors on most datasets, and our method outperforms the other methods in most cases. This validates the effectiveness of our framework. Take Dolphins dataset for example, NMI and Purity of our method can reach 1 with 10% priors, while NMF_LSE and PMF needs 15% priors, and even with 30% priors SNMF_SS do not have better performance.

### Parameter study

**Fig 4:**
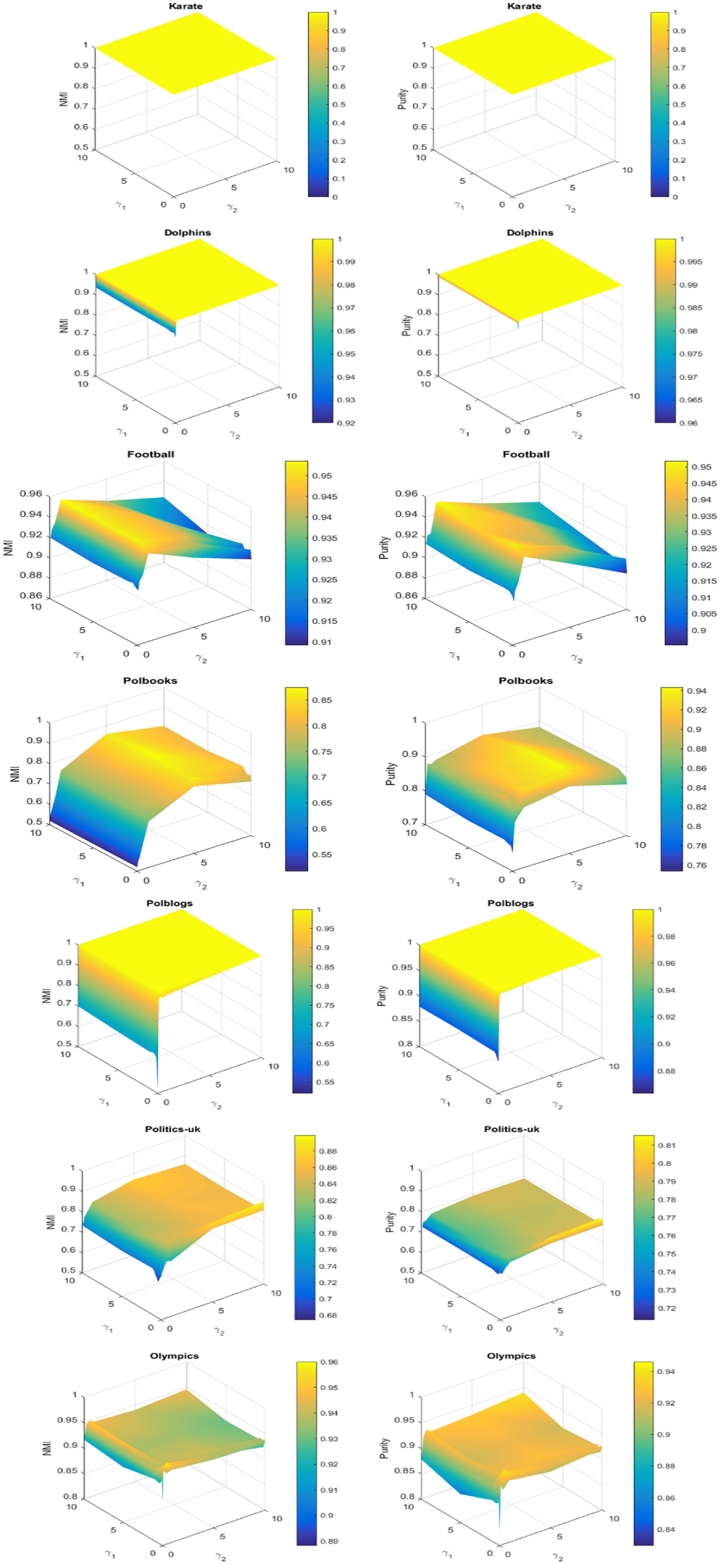
Experimental results of our algorithm with respect to parameters *γ*_1_ and *γ*_2_. In the experiments, 5% must-link priors and 5% cannot-link priors are added.

To illustrate the effect of the parameters and discuss how to determine them, we evaluate the role of balancing parameters. In Fig 4, we plot the performance of our algorithm as *γ*_1_ and *γ*_2_ vary from 0 to 10 with 5% must-link priors and 5% cannot-link priors on seven networks. We can observe that the value of NMI or Purity at point (0,0) is much lower than that at other points on all the datasets except Karate, then the performance improves much when there are priors. We can find that the performance remains relatively stable across a wide range of *γ*_1_ on most datasets, that is our method is not sensitive to parameter *γ*_1_. Then *γ*_1_ can be set to [0.1,10] to achieve a good performance. We can also observe that performance changes greatly with varying *γ*_2_ on most datasets, that is the method is sensitive to *γ*_2_. On Football, Polbooks and Politics-uk datasets, NMI and Purity first increase significantly, then decrease especially when *γ*_2_ > 5. On Dolphins and Polblogs datasets, NMI and Purity first increase as *γ*_2_ increases and then remains stable when their values reach 1. Specially, on Olympics dataset, the performance is much better than that without priors and remains relatively stable when *γ*_2_ > 0.2. Overall, from the figures, we can conclude that the algorithm has a good performance across a range from 1 to 5 of *γ*_2_. Furthermore, we can observe that on many datasets, the performance at point (1,1) is not the best. In the experiments of last section, *γ*_1_ and *γ*_2_ of our method are both set to 1, it outperforms all baseline methods on most dataset with varying percentages of priors. Then we can find that the best performance of our method is much better than those of the baseline methods. Based on experimental results, we suggest that *γ*_1_ and *γ*_2_ be set to [0.1,10] and [1,5] respectively to achieve a good performance.

## Conclusions

In this paper, we propose an efficient semi-supervised community detection framework based on NMF. In the framework, the must-link and cannot-link priors are transformed into positive and negative links respectively, and are encoded by two graph regularization terms to penalize closeness of the nodes. The proposed framework incorporates the pairwise constraints knowledge into the detection process seamlessly. Experimental results on real-world datasets demonstrate the effectiveness of our method. For the future work, we plan to explore how to utilize more types of prior information to guide the detection process.
